# Manifestations of Psychological Distress in Emerging Adults Experiencing Quarter-life Crisis: a Systematic Review

**DOI:** 10.1192/j.eurpsy.2026.10460

**Published:** 2026-07-31

**Authors:** J. P. Miyake, A. C. Barbosa, V. C. dos Santos, R. A. Passos, J. G. Tostes

**Affiliations:** Medicine, Faculty of Medicine of Itajubá, Itajubá, Brazil

## Abstract

**Introduction:**

Quarter-life crisis is a period of developmental adversity characterized by feelings of confusion, uncertainty and instability during emerging adulthood (ages 18-30). Despite its prevalence, it remains underexplored, making it essential to understand its psychological consequences.

**Objectives:**

This systematic review aims to review the manifestations of psychological distress within emerging adults experiencing the quarter-life crisis.

**Methods:**

This review was conducted following the PRISMA 2020 guidelines, and its abstract was prepared according to the PRISMA 2020 for Abstracts Checklist. Eligible studies investigated quarter-life crisis in individuals aged 18–30 and reported psychological outcomes (e.g., anxiety, stress). Search terms included emerging adulthood, quarter-life crisis, psychological distress, and related concepts, combined with Boolean operators. We included primary quantitative, qualitative, or mixed-methods studies published in peer-reviewed journals since January 1, 2015. Only English or Portuguese articles were considered; no Portuguese studies were retrieved. Grey literature was excluded except for one illustrative case report. Searches were performed in PubMed/MEDLINE, PsycINFO, Web of Science, Scopus, and CINAHL (last accessed September 2, 2025); Taylor & Francis Online and SpringerLink were searched to capture studies not indexed elsewhere. Study quality was assessed using the JBI Critical Appraisal Checklist for Qualitative Research and for Analytical Cross-Sectional Studies and the MMAT. Findings were narratively synthesized. PROSPERO ID: 1142550.

**Results:**

Six studies including 3,927 participants met inclusion criteria. Most studies originated from the United Kingdom and the United States, with one each from Canada and the Czech Republic. Psychological manifestations were categorized (Image 1) into emotional-existential distress (anxiety, depression, stress, low self-esteem, hopelessness, uncertainty, inner dissatisfaction, disillusionment, entrapment, unpreparedness, disorientation) and relational-expectational distress (loneliness, unfairness, failure, worry about the future, unmet expectations, disappointment). Anxiety was the most frequently reported outcome.

**Image 1:**
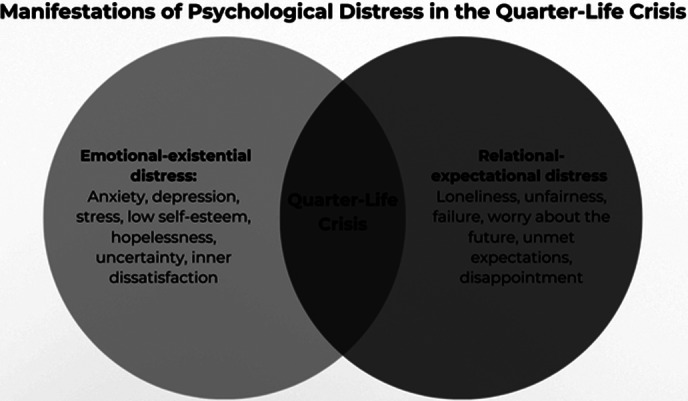
Image 1: Long description.

**Conclusions:**

Major life events during emerging adulthood, such as graduation, moving out, and career exploration, were linked to psychological distress. Older students reported lower anxiety and depression than younger peers. Limitations include the small number of studies, reducing generalizability, and the lack of research from developing countries, which restricts cross-cultural applicability.

**Disclosure of Interest:**

None Declared

## Long descriptions

### Image 1: Long description

The diagram consists of two overlapping circles.

* The left circle is light gray and labeled Emotional-existential distress. It lists the following symptoms: Anxiety, depression, stress, low self-esteem, hopelessness, uncertainty, and inner dissatisfaction.

* The central intersection is dark gray and labeled Quarter-Life Crisis, indicating the convergence of both distress types.

* The right circle is medium gray and labeled Relational-expectational distress. It lists the following symptoms: Loneliness, unfairness, failure, worry about the future, unmet expectations, and disappointment.

Navigate back to Image 1:.

